# The Child Protection Response to Domestic Violence and Abuse: a Scoping Review of Interagency Interventions, Models and Collaboration

**DOI:** 10.1007/s10896-024-00681-4

**Published:** 2024-02-19

**Authors:** Hannah Hale, Kelly Bracewell, Laura Bellussi, Ruth Jenkins, Joanne Alexander, John Devaney, Jane E. M. Callaghan

**Affiliations:** 1 Centre for Child Wellbeing and Protection, University of Stirling, Stirling, UK; 2 The Connect Centre, University of Central Lancashire, Preston, UK; 3 School of Social Work, Care and Community, University of Central Lancashire, Preston, UK; 4 Library and University Collections, University of Edinburgh, Edinburgh, UK; 5 School of Social and Political Science, University of Edinburgh, Edinburgh, UK

**Keywords:** Domestic abuse, Intimate partner violence, Scoping review, Interagency collaboration, Child protection, Multiagency working

## Abstract

**Purpose:**

There is a growing acknowledgement that children are direct victims of domestic violence and abuse, and require support and protection in their own right. However, professional interventions designed to protect children may unintentionally further victimise parents, most often mothers. In response, a number of new interagency approaches have been developed.

**Method:**

Updating a previous review by Macvean et al. (*Australian Social Work,* *71*(2), 148–161, 2018), we report the findings of a scoping review of models of interagency working between child protection and either domestic abuse services or family law services, or all three services, to improve understanding of practices that may facilitate collaboration between child protection and other agencies in the context of domestic violence and abuse. We also consider the effectiveness of such approaches in improving the safety of child and adult victims.

**Results:**

A systematic search of all sources identified 4103 documents that were screened for inclusion. The outcome of this screening was the identification of thirteen papers or reports dated between 2018 and 2022 that comprised an evaluation of six models of interagency interventions. Nine publications originated in Australia, three in the UK and one in the USA. The most referenced model was Safe & Together, primarily due to the number of publications from the same research team in Australia. None of the included studies reported the outcomes or impact for children and families.

**Conclusions:**

While there are a growing number of promising approaches identified, there is little evidence of effectiveness, or the views of child and adult family members about the acceptability and utility of such approaches.

**Supplementary Information:**

The online version contains supplementary material available at 10.1007/s10896-024-00681-4.

## Introduction

Despite the lack of official statistics in many countries on the prevalence of childhood exposure to domestic violence and abuse^1^, there is growing research evidence suggesting that it is a widespread and common phenomenon (for example, Chan et al., 2021; Kieselbach et al., 2022; Mojahed et al., 2021; Skafida et al., 2022). Studies in high-income contexts estimate that the prevalence of domestic violence and abuse in childhood is between 20 and 31% (Loomis et al., 2020; Radford et al., 2011). There is also evidence that the Covid-19 pandemic has increased the incidence of domestic violence and abuse for adult and child victims (Kourti et al., 2023).

There is a growing acknowledgement that children are direct victims of domestic violence and abuse and require support and protection in their own right (Callaghan et al., 2018), with the Domestic Abuse Act 2021 in England and Wales recognising this distinct status. However, there is also concern that professional responses aimed at keeping children safe might also have the unintended consequence of further victimising parents, most often mothers (Arnull & Stewart, 2021; Cattagni Kleiner & Romain-Glassey, 2023), through what is termed a ‘failure to protect’ discourse (e.g. Buchanan & Moulding, 2021; Friend et al., 2008; Moulding et al., 2015). Since abusive partners are less likely to engage with professionals (Heron & Eisma, 2021), social work professionals are often left with only the non-abusive partner to work with. This can be compounded by professionals’ lack of confidence and skills to engage with violent men and to hold them accountable for the risk they pose to their current or former partners and their children (Devaney, 2014). The default position for many professionals is to place responsibility for keeping children safe on the children’s mother (Buchanan & Moulding, 2021; Cattagni Kleiner & Romain-Glassey, 2023).

A range of interagency models and ways of working have been developed to reduce victim blaming practices and improve child protection responses for both adult and child victims of domestic violence and abuse. These models aim to equip practitioners with the knowledge and skills to engage with families in ways that are sensitive to the dynamics of domestic violence and abuse, recognising the particular importance of keeping children safe. These models aim to enable whole-family working that recognises the specific needs of adult and child victims, whilst also holding accountable individuals who engage in controlling, abusive and violent behaviour (Stewart & Arnull, 2023).

Previously, Macvean and colleagues (2018) undertook a scoping review of models of interagency working between child protection and either domestic abuse services or family law services, or all three services, to improve understanding of practices that may facilitate collaboration between child protection and other agencies in the context of domestic violence and abuse. Our review seeks to update Macvean et al. (2018) with the research published from the end of the period of the first review (April 2015) to July 2022, with a particular focus on identifying outcomes for adult and child survivors, and perpetrator engagement.

The original review (Macvean et al., 2018) identified and described the key features of multi-agency approaches that were focused on how services could work together toward improved responses for women and children. These approaches were characterised by:

- a shared understanding within and between services of the nature and causes of domestic violence and abuse, expressed in the overarching aims and goals of the co-operating organisations. Typically, the models used a socio-ecological approach to shape interagency collaboration and to inform the design and delivery of services.
- clear leadership to guide interagency working. This could be delivered through, for example, a shared governing committee and joint budgets.
- an authorising environment, that included formal management ‘buy in’ to the model, as well as a supportive infrastructure, including, for instance, shared funding and professional development opportunities, joint training, common procedures, and clear mechanisms for quality assuring the joint work.
- formal agreements and shared protocols to facilitate interagency collaboration. This could be supplemented by the co-location of practitioners or teams, joint training, and joint working.
- an organisational commitment to information-sharing, including practical means of sharing of information that would facilitate joint working between different agencies with different mandates, such as which family member they were primarily working with.

In seeking to update and extend the previous review, our main research question, in keeping with the original review, is:

1. What processes or practices do child protection services and specialist domestic violence services or family law services utilise to improve interagency collaboration in responses for women and children living with and separating from domestic violence and abuse?

In addition, we seek to extend the original review by having two supplemental objectives, which seek to identify:

2. The types of interventions and their impact on professionals’ understanding and responses to women, children and men upon disclosure of domestic violence and abuse, or of disclosure of child maltreatment in the context of domestic violence and abuse; and
3. The evidence of effectiveness in how organisations have responded to domestic violence and abuse in the context of child protection.

## Method

A scoping review methodology was used to locate and examine evaluations of collaborative models in which child protection, domestic abuse services and family law services worked together. The aim of a scoping review is to use rigorous and transparent methods to identify studies on specific topics that little is known about (Arksey & O’Malley, 2005).

### Selection Criteria

Studies using any evaluative design were included if: they were published since May 2015; they were published in English language; they comprised an evaluation of a model in which child protection services and domestic abuse services, or child protection services and family law services, or all three worked together; the focus of the evaluation is on male violence perpetrated against women; they were reporting explicitly on the potential benefit of the model for children aged 0 to 18 years; and they comprised any outcome measure that reported the impact of interagency work on some form of child, parent, family, service-provider, or organisation outcome.

Studies were excluded if: they did not report original findings; they were published before May 2015; they were not published in the English language; they were an evaluation of a model that only applies to or involved one service; if they did not report an evaluation of a model, for example, a study assessing the interface or extent of collaboration between sectors, services, or providers, without an identified arrangement for interagency working; there was no clear indication of child protection services involvement; the focus of the literature was on violence perpetrated in same-sex relationships, trans relationships or female perpetrated violence; they comprised studies on domestic violence and abuse where there is no child aged 0 to 18 years in the family or home; or they comprised studies addressing elder abuse, carer abuse, sibling abuse, and child to parent violence.

For the purposes of this review a model is any formal intervention that requires practitioners to follow certain ways of working that are prescribed, and where an agency would need to give formal approval to the adoption of that way of working.

### Search Methods

#### Electronic Database Searches

The following databases were searched from May 2015 to July 2022: PsycINFO via Ovid; MEDLINE® via Ovid; Embase via Ovid; Cumulative Index to Nursing and Allied Health Literature (CINAHL) via EBSCOhost; Criminal Justice Abstracts via Ebsco host; Education Resources Information Centre (ERIC) via Ebsco host; Applied Social Sciences Index and Abstracts (ASSIA) via Proquest; Sociological Abstracts via Proquest; Social Care Online and Social Work Abstracts via Proquest. These databases were chosen as they indexed journals covering key areas of interest to this review.

Search terms were used in Ovid Databases, with adaptations for ProQuest and EBSCO as needed. Searches were downloaded in July and August 2021 and updated in July 2022,

#### Searching Other Sources

In addition, published sources of literature were sought from the following online sites: the New Zealand Family Violence Clearing House; the National Clearinghouse on Family Violence; the Centre for Research and Education on Violence Against Women and Children: Australia’s National research Organisation for Women’s Safety (ANROWS); Australian Institute of Family Studies - Child Family Community Australia; the Child Protection Research Centre; and the National Child Traumatic Stress Network. References and citations in relevant literature according to the search criteria were screened for documents that may have identified further model details and findings.

### Data Collection and Analysis

#### Selection of Studies

All results from electronic database searches were exported to the bibliographic software programme Endnote and then to Covidence (a systematic review management tool). Duplicates were removed. An initial 20% of titles and abstracts were screened independently by researchers. Disagreements were few and were resolved following a discussion of selection criteria that enabled the team to agree on final decisions. Once the team were comfortable with the operationalisation of our selection and exclusion criteria, the remaining 80% of titles and abstracts were screened by three members of the team (HH, KB and JA) with a sample of both included and excluded literature randomly reviewed by a separate team member (JD).

#### Data Extraction, Analysis, and Synthesis

Full texts of results were read by three researchers (HH, KB and LB). A proforma was used for consistency in data extraction. This then allowed the research team to synthesise the findings from across the included literature.

## Findings

### Search Results

**Fig. 1:**
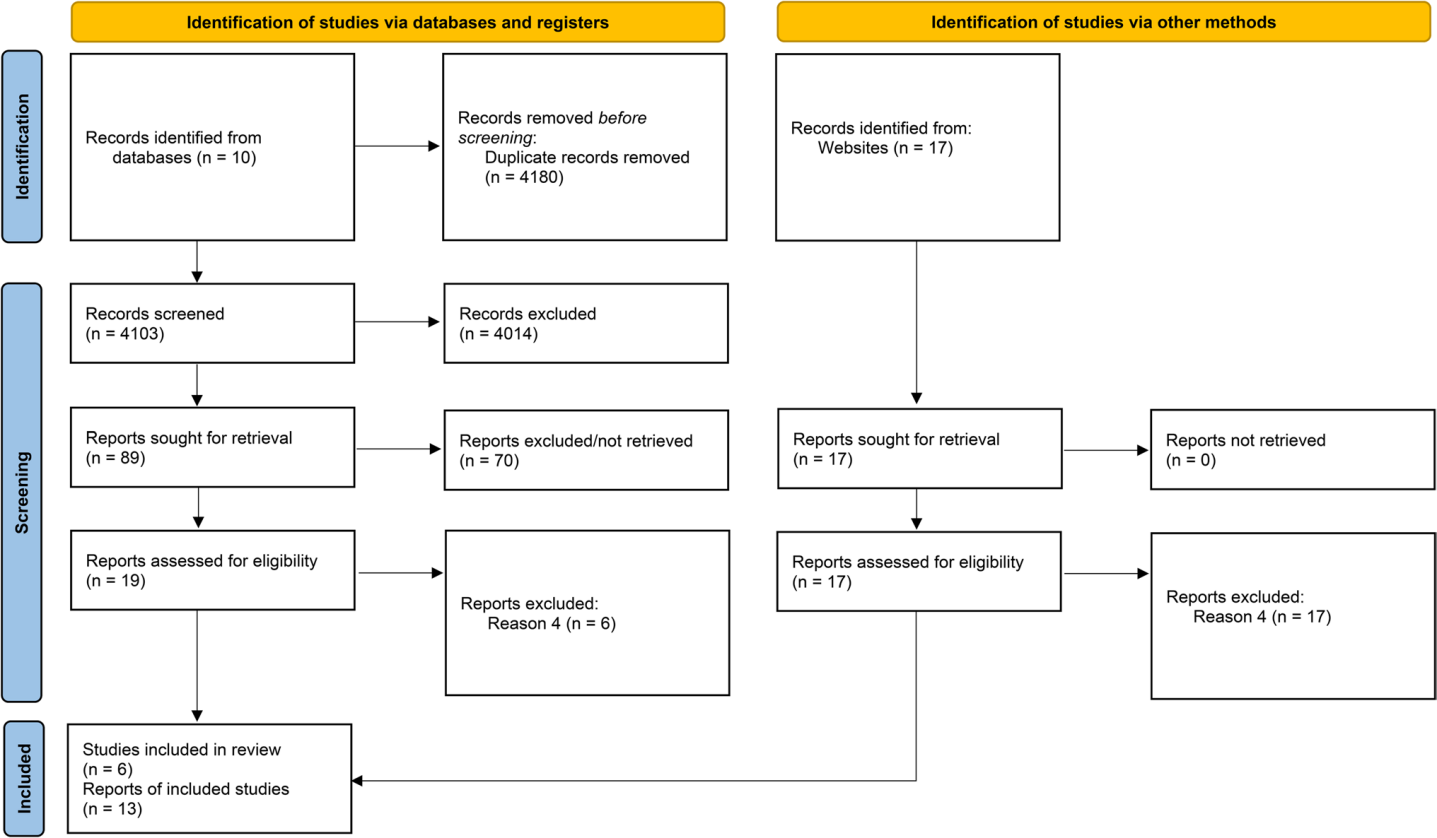
PRISMA diagram of included studies. Exclusion Criteria: Reason 1: *Published before May 2015.*. Reason 2: *Not published in English language.*Reason 3: *An evaluation of a model that only applies to or involves one service.* Reason 4: *Does not report an evaluation of a model, for example, a study assessing the interface or extent of collaboration between sectors, services, or providers, without an identified arrangement for interagency working, would be excluded.* Reason 5: *Models will be excluded if there was no clear indication of child protection services involvement.* Reason 6: *The focus of the review is on violence perpetrated in same sex relationships, trans relationships or female perpetrated violence.* Reason 7: *Studies on domestic abuse where there is no child aged 0 to 18 years in the family or home.* Reason 8: *Studies addressing elder abuse, carer abuse, sibling abuse, violence outside the domestic abuse context, and adolescent violence towards parents*

 A systematic search of all sources identified 4103 documents that were screened for inclusion. The outcome of this screening was the identification of thirteen papers or reports dated between 2018 and 2022 that comprised an evaluation of six models of interagency interventions. Of these, nine publications originated in Australia, three in the UK and one in the USA (Fig. 1).

Of the included work (Table 1), eight were qualitative studies, and five used a mixed methods approach. Qualitative and mixed methods studies made use of interviews, focus groups, ethnographic data – mostly from observation of “Communities of Practice”, case file analysis, analysis of administrative data, and questionnaires, as data collection methods. No formal and structured assessment of perpetrator behaviour change was undertaken.

**Table 1:** Included studies

Study	Country of Study	Model and Aim of Study	Method	Key findings
Bostock et al. (2018). Diffusion theory and multi-disciplinary working in children’s services. *Journal of Integrated Care*, 26(2), 120-129.	UK	Model: Multi-disciplinary children’s safeguarding team (early help) Aim: To explore how innovation in children’s services is adopted and developed by staff within new multi-disciplinary children’s safeguarding teams.	Interviews with 61 frontline safeguarding staff, including social workers, substance misuse workers, mental health workers and DA workers. Thematic analysis identified the enablers and barriers to implementation.	- Diffusion of innovations (DOI) theory defines five essential innovation attributes: relative advantage over current practice; compatibility with existing values and practices; complexity or simplicity of implementation; trialability or piloting of new ideas; and observability or seeing results swiftly. - Multi-disciplinary team working and group supervision is advantageous, in line with social work values and improved their service to children and families. - Motivational interviewing and new ways of case recordings were less readily accepted because of the complexity of practicing confidently and concerns about risks of moving away from exhaustive case recording.
Heward-Belle et al. (2019). Invisible practices: Interventions with men who use violence and control. *Affilia*, 34(3), 369-382.	Australia	Model: Invisible Practices Project Aim: Designed to explore the practices of workers who see perpetrators of DA outside the relative safety of group-work programs. This article reports data from The Invisible Practices Project: Engaging With Fathers Who Use Violence. It investigates the organizational context that contributes to, or constrains, the practices of frontline workers intervening with fathers who use violence.	Involved a range of methods in the wider study. Data from this article are derived from opened-ended responses to questionnaires from 232 frontline workers and team leaders from a range of organizations across four Australian states (in the Invisible Practices Project).	- When practitioners chose to see and hear domestically violent men, opportunities arose publicly legitimize women’s and children’s experiences. - Visibility differs within and across agencies and is a crucial driver orienting their engagement in policy and practice. - Engaging fathers in discussions about violence for risk assessment purposes was the most commonly reported development. - Other emerging practices included engaging in conversations about fathering experiences and practices. - Bearing witness requires a legal and service system that promotes survivor safety, honours their resistance, holds perpetrators accountable, and constructs them as capable of change. - Governance structures were associated with dangerous institutional practices. - Consequences of insufficient policy and funding arrangements, inadequate governance structures, limited professional supervision, and insufficient safety procedures to support complex work are highlighted.
Heward-Belle et al. (2020). Practice-led research: Developing communities of practice to drive research and practice change in the domestic and family violence arena. In *The Routledge Handbook of Social Work Practice Research* (pp. 418-429). Routledge.	Australia	Model: Safe & Together Aims: 1.How do workers, as part of case management, assess and manage the complexity of the intersections of MH, AOD and DFV while maintaining the DFV focus?2.What formal collaborative arrangements are required for workers and their organisations to intervene where DFV, MH and AOD intersect?3.In what ways does the S&T Model inform worker practice where there are issues of complexity?	Practice-led research (linked to larger STACY study). S&T Practitioners from different organisations across three different states in Australia meeting regularly over several months to receive training and participate in a series of six community practice groups and case discussion.	- Dual focus on collaborative learning and co-designed knowledge production through interrogation of complex cases enabled practitioners to think about their work, service users and the legal and social service system from a different vantage point. - This resulted in more nuanced understandings of the intersections between mental health, substance use and DFV. - The often-invisible perpetrator of violence was illuminated and the systematic secondary abuse of mothers through the interventions of different organisations was challenged.
Humphreys and Healey (2017). *PAThways and Research Into Collaborative Inter-Agency Practice: Collaborative Work Across the CP and Specialist Domestic and Family Violence Interface-the PATRICIA Project*. Sydney: ANROWS.	Australia	Model: Safe & Together Overarching research question: What are the elements that facilitate differential pathways and appropriate service system support for the safety and wellbeing of women and children living with, and separating from, domestic and family violence in an integrated intervention system with a specific focus on CP and specialist DFV services? Research aims: 1) provide a scoping review of the evidence specific to collaborative working in the fields of CP, family law, and DFV. 2) synthesise the evidence to identify the elements required to collaborate in working with CP, DFV, and family law services. 3) apply the identified criteria for collaboration across five case studies in different states of Australia to elucidate a rich understanding of the barriers and facilitating factors for collaborative working.4) use current data from state CP systems to understand the differential service pathways for a diverse group of women and their children living with DFV and how these differ from cases where DFV is not reported.5) apply the S&T case reading method to illuminate the strengths and problems in current statutory CP practice where there is DFV.6) provide recommendations for policy, systems, and service delivery improvements at the interface of CP, family law, and domestic violence systems.	The case study project included 5 sites (New South Wales, Queensland, South Australia, Victoria, and Western Australia). Each of the five state-based research teams investigated a current, positive collaborative initiative involving CP, specialist domestic and family violence services, and, wherever possible, family law. Multi-method approach included: interviews; secondary analysis of existing evaluation data; action research or observation; analysis of policy or practice documents.	- formalised governance arrangements that provide an authorising environment for collaboration between CP and DFV specialist services ensures that collaboration is built on more than particular personal relationships. - training alone is inadequate. Practitioner coaching (central to S&T practice change model) is required. - Policy work is required to support the development and implementation of S&T. - Problems and possibilities were identified for collaborative working: governance, management and operations, and quality monitoring. Management and operations included entry into the service system, service planning, service provision, and service array. - There is a lot of unidentified DFV in the analysed administrative data or a lack of differentiation between children living/ not living with DFV. This means DFV is not a primary factor in decision-making about out-of-home care. - Services, beyond criminal and civil justice responses, need to be available for fathers who use violence. - Information sharing provided a critical operational factor for collaborative action in terms of improving the DFV response. - Risk assessment and management tools assist practitioners to assess the type of intervention required to respond to a DFV incident. - A differential response ensures that children who do not reach the CP threshold for investigation are referred to (specialist DA) services. - Co-location was highly valued in the development of collaborative practice.
Humphreys et al. (2018a). Case reading as a practice and training intervention in domestic violence and child protection. *Australian Social Wor*k, 71(3), 277-291.	Australia	Model: Safe & Together Aim: To assess the extent to which DFV is identified in CP case files and to assess the quality of case practice from a DFV-informedperspective, as documented in the case file. This article explores the role of case reading, a tool developed by David Mandel to support more effective and sophisticated working where there are children living with DFV.	Analysis of 20 case files in 5 CP organisations, included 30 experienced professionals in the process (selected by managers in CP and specialist DFV services).	- The process of case reading is an important enabler for improving competencies in risk assessment, case decision-making, complex case planning, and cross-system collaboration. - Case-reading indicated that documented CP practice sits at the lower end of DA-informed Continuum of Practice. - Analysis indicates a need to improve: CP engagement with fathers who use DFV, assessments of their parenting role and its impact on children and family functioning; identification of adult survivors’ protective capacities and their impact on children. - Serious concerns were raised about the quality of DFV risk assessment, case decision-making, case planning, and case complexity.
Humphreys et al. (2018b). Children living with domestic violence: A differential response through multi-agency collaboration. *Australian Social Work*, 71(2), 162-174.	Australia	Model: Multi-Agency Triage Aim: To highlight the importance of collaborative interagency partnerships in responding to children, mothers, and fathers living with DA; and the impact of effective collaboration on the development of a differential response that diverts children early from a statutory response.	Case study of the Multi-Agency Triage project (Melbourne): semi structured interviews (n=9) with participants and their mangers; observations of the triage; and analysis of administrative data.	- Strong collaborations between organisations need to provide the foundation for differentiated referral pathways for children living with DFV based on informed risk assessment. - Through multi-agency risk assessment, children and their families who do not meet the CP investigation threshold can be diverted early into a community sector organisation. - A number of issues were found regarding partnership working and information sharing. - Leadership and an authorising environment are critical to collaboration. - Structures for governance and operation of the triage model supported development of effective relationships and systems.
Humphreys et al. (2020). *Safe & Together Addressing ComplexitY for Children (STACY for Children) (Research report, 22/20). *Sydney: ANROWS.	Australia	Model: Safe & Together Aim: The STACY for Children project (2019-20) involved two studies that investigated whether there was emerging evidence that the S&T Model, where it is implemented holistically, is leading to better outcomes for children and families living with domestic and family violence (DFV) and parental issues of alcohol and other drugs (AOD) use and/or MH problems. It was not the aim of this study to evaluate the effectiveness of S&T itself, but to conduct an exploratory analysis of the early impacts of the availability of S&T on CP process outcomes at the trial Child Safety Service Centre (CSSC).	Study 1 re-analysed data collected in the STACY project with a focus on children’s needs and perspectives. Secondary analysis enabled deeper understanding of practitioner perspectives on the implementation of the S&T Model. Study 2 explored quantitative data from CP in an area where the S&T Model had been proactively implemented. A variety of analytical approaches were applied including: descriptive analytics, pre/post analyses and non-equivalent comparison group designs.	As above. See: Australia’s National Research Organisation for Women’s Safety. (2020). Safe & Together Addressing ComplexitY for Children (STACY for Children): Key findings and future directions (Research to policy and practice, 22/2020). Sydney: ANROWS
McCarry et al. (2021). What Helps? Mothers' and Children's Experiences of Community‐Based Early Intervention Programmes for Domestic Violence. *Child Abuse Review*, 30(2), 114-129.	UK	Model: Safer Together Aim: To understand what is effective in early help or early intervention for safeguarding children living with DA.	Investigation of qualitative data gathered from an evaluation of a new early help service for children living with DA. Data was gathered from 39 participants (mothers, children, service providers) via interviews and focus groups. Plus referral data and entry/ exit outcomes.	- Substantial cultural shifts in practice are required to move from a reactive and crisis-focused response towards DA to an early intervention and more preventive approach. - It can be a very challenging task to gather evidence on the effectiveness of early intervention for children and adult victims/survivors living with DA. - Children, mothers and service providers reported both a perceived need for early help and a positive impact from DA early help services on child health and emotional wellbeing. - flexibility of key worker-led service delivery to the needs of families is important for supporting engagement of, and fostering a sense of control for, families receiving support. - Confidentiality, reliability, respect and trust are key factors in developing an effective key worker-family relationship.
O’Leary et al. (2018). Interagency working in child protection and domestic violence. *Australian Social Work*, 71(2), 175-188.	Australia	Model: Gold Coast Domestic Violence Integrated Response (GCDVIR) Aim: To better understand how CP agencies and DA services approach collaboration, and may assist social workers to improve practice, enhancing the safety of women and children.To establish what facilitated effective service responses that support the safety and wellbeing of women and children living with and separated from DA.	Sample included 30 professionals from a range of agencies, including DA, CP (almost half), and justice services. Qualitative data from semi-structured interviews was analysed thematically.	- Progress was evident in the operation of the model with trust built through collaboration. - Differing mandates created tensions between DFV and CP sectors. - Gaps in understanding and a reliance on informal relationships were most evident amongst front-line workers. - A model was developed for measuring integration within inner and outer circles of the GCDVIR. - Social work has a key role in creating a dynamic model that is able to critically evolve to meet safety needs of women and children experiencing DA.
Sen et al. (2018). ‘When you're sitting in the room with two people one of whom… has bashed the hell out of the other’: Possibilities and challenges in the use of FGCs and restorative approaches following domestic violence. *Children and Youth Services Review*, 88, 441-449.	UK	Model: Family Group Conferencing Aim: To evaluate the use of Family Group Conferences (FGCs) in one Local Authority. This draws from data gathered from an evaluation of a UK Government funded “Innovation Project” which extended the use of FGCs in an urban local authority area already making extensive use of them. The development of a typology of FGCs was a key finding from the evaluation. This paper presents and explores this typology used in situations of DA: pragmatic, resolution-focussed and restorative FGCs.	FGC service evaluation was mixed method and multi-modal, taking place over 8 months. Methods included: analysis of administrative service data; practice observations; 10 case studies of families; focus groups with different groups of co-ordinators at three time points; semi-structured interviews (n=39); questionnaires (n=66) with FGC co-ordinators and managers; and structured telephone interviews with adult family members (n=36) referred due to concerns about DA, but not exclusively.	- pragmatic FGCs were the most used, restorative the least. Each FGC type brings potential benefits; only restorative FGCs offer the possibility of full restoration. - Present mother-centric, risk-adverse, CP systems provide a powerful resistor to implementation of restorative FGCs. - A pre-condition for a DA perpetrator's involvement in a restorative process is their willingness to take responsibility for harm. This prevents restorative FGCs taking place. - Perpetrator involvement is restricted by routine social work practices i.e. responses which determine families must separate and violent men are too risky to be engaged. - Greater realism is needed about the on-going involvement of perpetrators in children's lives, and greater effort to engage paternal networks and perpetrators in restorative discussion at an early stage. - FGCs can be used restoratively to reduce violence while foregrounding safety concerns for women and children. - Extensive engagement and preparation with families, support for families, provision of preventative DA services and, beginning to unpick the structural and power inequalities underlying DA, are all necessary to making restorative approaches work well.
Stevens et al. (2019). Detecting and reducing post-traumatic stress among children exposed to domestic violence: A multi-agency early intervention program. *Children and Youth Services Review*, 101, 261-269.	United States	Model: Child Trauma Response Team Aim: To examine the reach of CTRT (Child Trauma Response Team) services, and articulate the lessons learned in the early stages of CTRT program implementation. Describe the children and families served by CTRT (e.g., gender, race/ethnicity, age)Describe the lessons learned in the early stages of CTRT program implementation, including challenges and how they were addressed or overcome.	Mixed-methods study. Analyzed Child Trauma Response Team administrative data along with qualitative stakeholder interview data	- The majority of children accessing CTRT were young and came from racial and/or ethnic minority backgrounds. - Vast majority of families received safety assessment and planning and child trauma education, with many families receiving at least one other service. - Implementation challenges included: identification of eligible families; initiating and ensuring program engagement; and collaboration and communication across agencies. - Through inter-agency collaboration, reaching and serving children exposed to DA in the days and weeks immediately following a violent event is feasible - even in a large city with complex inter-agency relationships.
Tsantefski et al. (2018). Inclusivity in interagency responses to domestic violence and CP. *Australian Social Work*, 71(2), 202-214.	Australia	Model: Gold Coast Domestic Violence Integrated Response (GCDVIR) Aim: What are the elements that facilitate differential pathways and appropriate service system support for the safety and wellbeing of women and children living with and separating from family violence in an integrated intervention system?The article focuses on the workings of a well-established integrated response in relation to Minoritised populations.	30 staff (ranging from front-line workers to regional managers) from member organisations of the integrated response participated in semi-structured interviews.	- Service providers were cognisant of Minoritised populations. However, poor recognition of their and challenges in incorporating cultural and DA knowledge inhibited engagement. - Enhanced collaboration between agencies and self-reflexivity in a culturally responsive approach may assist in assimilating narratives of racism, colonialism, and sexuality into integrated responses’ core philosophies.
Tsantefski et al. (2021). High-risk cases at the intersection of domestic/family violence and CP: learning from practice. *Journal of Family Violence*, 36, 941-952.	Australia	Model: Safe & Together Aim: To understand what practitioners require from their organisations and/or other organisations to support them in working with fathers who use violence To understand if the capacity building of CoPs, supported by coaching and supervision by the S&T Institute, provides increased experiences of safety and support for practitioners. Conducted as part of the Invisible Practices: Intervention with fathers who use violence project, funded by Australian National Research Organisation for Women’s Safety (ANROWS).	This paper reports on qualitative research conducted with practitioners from a range of justice and service delivery organisations in Queensland. A community of practice, supported by the US based S&T Institute, provided the forum for data to be collected on participants’ reflections and observations. An action research framework was employed to connect practice learning into research data through an iterative cycle of reflection and review. This paper reports the results from 15 participants (12 women and 3 men) who contributed to Community of Practice (CoP) workshops.	- Four key themes concerning risk assessment and safety planning in high-risk DFV CP cases emerged: learning from and partnering with women; applying a perpetrator pattern approach; engaging with men as fathers; and the role of the judicial system. - practice needs to be informed by centring the victim/survivor perspective, which requires collaboration with women, not coercion. - workers are safer when they incorporate women’s voices, which is only possible through partnering with women and fully understanding the daily steps they take to keep their children and themselves safe. - relationships with children are not a motiving factor for behaviour change among the most dangerous men. - lack of communication and collaboration between service sectors and the courts hampers wrap-around support for women and children and increasing risks to worker safety. - Training in the S&T model for all judiciary will promote a more informed and integrated system for managing high risk cases. - Children were absent in practice discussions on high-risk cases.

### Key Themes

This scoping review aimed to refresh the Macvean et al. (2018) study and to highlight any new research findings that explicitly addressed the impact of the models identified. Findings, therefore, describe the types of interventions and their impact on professionals’ responses, summarise findings on the effectiveness of the models, and particularly highlight reported impacts for adult and child victims.

### Models of Intervention Evaluated in the Reviewed Studies

In relation to our first research question, the reviewed literature described several innovative collaborations between child protection services and specialist domestic violence and abuse and/or family law services. The most referenced model was Safe & Together (https://safeandtogetherinstitute.com/). The Safe & Together model is defined as an approach to domestic violence and abuse practice “that provides guidance for statutory CP [child protection] intervention as well as guidance for other services that not only engage with perpetrators as parents but offer multi-dimensional services to the whole family – perpetrator, adult and child victims/survivors” (Healey et al., 2018). The Safe & Together model centres its practice around a shift of practitioners’ language towards a more compassionate conceptualisation of the role of the non-abusive parent and increased accountability for the abusive parent’s role (Healey et al., 2018). The model primarily involves the training of practitioners and use of specially developed resources. While the model is currently used in several countries, all the literature on Safe & Together in this review (n = 5) was from Australia (Heward-Belle et al., 2020; Humphreys and Healey, 2017; Humphreys et al., 2018a, b, 2020; Tsantefski et al., 2021).

The Gold Coast Domestic Violence Integrated Response was described in two Australian articles (O’Leary et al., 2018; Tsantefski et al., 2018). Based on the Duluth model, this is a co-ordinated multi-agency approach to women and children affected by domestic violence, and to men who perpetrate violence. Involving fifteen agencies, the approach comprises monthly meetings, and twice-weekly triage meetings, to manage high-risk cases, ongoing information sharing, and men’s behavioural change programs for perpetrators. It aims to provide wrap-around support for women and children while holding perpetrators accountable and managing risk.

Two articles from the UK analysed the impact of different early help services in different localities, designed to be part of a holistic care response for children and mothers within an existing consortium of specialist domestic violence and abuse services. These multi-disciplinary family safeguarding teams involved substance abuse and mental health practitioners and domestic abuse specialist workers, alongside social work practitioners (Bostock et al., 2018; McCarry et al., 2021). Another UK-based study explored the features of different types of Family Group Conferences based on the principles of restorative justice applied in cases of domestic violence and abuse (Sen et al., 2018).

In the United States, an innovative multi-agency collaboration called Child Trauma Response Team (CTRT) aimed to provide coordinated, immediate, trauma/survivor-informed, and interdisciplinary responses to children and their family members who are exposed to domestic violence and abuse (Stevens et al., 2019).

The remaining two models were both from Australia. The Invisible Practice Project was an action research study designed to explore the practices of workers who see perpetrators of domestic violence and abuse outside the strictures of group-work programmes (Heward-Belle et al., 2019). The Multi-Agency Triage involves collaboration between child protection services, family services, an Aboriginal children’s organisation, and the separate specialist domestic and family violence services for women and men to ensure stronger and more effective case management of children living with domestic and family violence (Humphreys et al., 2018b). The approach involves a “rapid” triage based on information from the different organisations’ databases and the police referral to assess the risk and determine the appropriate service pathway for engagement with the adult victim-survivors, children, and perpetrators.

### How Do These Interventions Impact on Improving Professionals’ Assessment of and Responses to the Disclosure of Domestic Violence and Abuse?

Our second research question inquired whether such innovations have an impact on practitioners’ understanding and response to disclosures of domestic violence and abuse. This outcome was, at times, challenging to disentangle from broader descriptions of process reported in the studies. Examining the impact of interventions on professionals’ responses to domestic violence and abuse in child protection, the literature focuses on several areas: how interventions might improve practitioner knowledge and understanding of domestic abuse; how they contribute to a more critical understanding of the role of social and structural issues like culture, race and gender in domestic violence and abuse; and how training and service transformation helped to make practitioners more focused on the role of the perpetrator, and more focused on the protective efforts of the victim-survivor.

#### Practitioner Knowledge and Understanding

The literature reviewed suggests that interagency collaborative models are effective in increasing knowledge and understanding of domestic violence and abuse, and based on practitioner self-report and case file audit, it seems that both attitude change and changes in practice are achievable. For example, Tsantefski et al. (2021) reported that, in Safe & Together Communities of Practice, workers in child protection pointed out contrasts between their pre-training assumptions about women’s attitudes, and the way these had transformed through their experiences of training and engagement with the communities. They highlighted that their previous assumptions had led to punitive professional decision-making and described ways that they had modified their everyday practice accordingly. The study by Humphreys and colleagues (2020) reported that the disciplinary background of the practitioners influenced how they experienced Safe and Together training. For instance, mental health and substance use professionals in the collaboration had lower baseline confidence in personal and organisational practice around domestic violence and abuse than specialist domestic abuse workers and social workers did. However, professionals working in substance use, social work and domestic violence and abuse services all reported significant changes as a result of training. They also experienced meaningful change particularly in their understanding of how child safety and wellbeing are tied to those of the adult victim-survivor. In contrast, mental health services were reportedly less responsive to change.

It was noted however, that further training was needed to enhance practitioner understanding of the role of drugs and alcohol, particularly the ways that substance use and mental health issues are used as part of wider patterns of coercive control, and this requires training and focus (Humphreys et al., 2020).

#### Understanding the Intersections of Domestic Violence and Abuse with Structural Inequalities and Discrimination

Two papers discussed the importance of practitioner understanding of diversity and the intersections of culture and gender in the experience of domestic violence and abuse. They suggested that practitioners’ understanding of cultural issues would impact on their response to domestic violence and abuse. Whilst “A Safe & Together practice guide” in Australia recommended that clients should be matched with practitioners of the same cultural background, other studies suggested that practitioners should treat all clients as equals without making distinctions between cultures, arguing that minority communities might fear being judged by their own group’s dominant cultural standards (Tsantefski et al., 2018). The evidentiary basis for both positions is not particularly clear but does highlight the importance of cultural sensitivity and awareness of how power structures, such as racism, can intersect and have an influence on abusive behaviour (Tsantefski et al., 2018).

Heward-Belle et al. (2020) found that Safe and Together training enhanced participants’ critical understanding of the social structures that informed policy and practice, and challenged individualising and pathologising preconceptions about domestic violence and abuse. This enabled social workers and other practitioners working in social work to identify how they had become enculturated in specific organisational contexts that perpetuated sexist and racialised biases in policies and practices. Working together as a community of practice, practitioners took action to advocate for more effective organisational responses that did not hold women accountable for the consequences of men’s violence. In contrast,Tsantefski et al. (2018) reported mixed findings when examining the culturally appropriate and inclusive nature of child protection work in responding to domestic violence and abuse, and suggested that largely, practitioners were using culture ‘neutral’ approaches, and focused on treating clients equally and avoiding discrimination, rather than understanding how culture might shape their clients’ experiences and needs.

#### Partnering with Non-Abusive Parents and Pivoting to the Perpetrator

Tsantefski et al. (2021) suggested that an *acknowledgement* of women’s safety planning was an important step in partnering with victim-survivors and in managing risk. Child protection workers adopted some of the same strategies women used to ensure their safety, and that of their children, to manage their own anxieties and safety. Practitioners still reported fear and unpreparedness in engaging with fathers. While the application of a perpetrator pattern-based approach was considered essential for determining and sharing understanding of the level of risk for women and children, the emergent pattern could exacerbate worker anxiety.

Heward-Belle et al. (2019) found that Safe and Together’s ‘Invisible Practices’ approach to working with fathers who use violence and control built practitioners’ confidence in working with people who behave abusively, by developing techniques and strategies that enabled them to more effectively ‘pivot to the perpetrator’. The strategies for keeping the perpetrator of domestic violence and abuse in view, and for understanding the ways in which substance use and mental health issues are used as part of wider tactics of coercive control, require training and focus. This requires embedding practice that moves beyond identifying the co-occurrence of these issues towards an understanding of how they are intersecting and connected (Humphreys et al., 2020). Practitioners highlighted the value of mapping out the different forms of harm the perpetrator posed, as this enabled them to transform their practice away from focussing on the mother’s supposed failure to protect, onto the harmful parenting choices of the perpetrator. They reported that strategies for keeping perpetrators visible could enable practitioners to more fully conceptualise how survivor’s alcohol and drug use and mental health issues might be weaponised by the perpetrator as tools of coercive control. However, they noted that this required fuller training and focus, to enable practitioners to move beyond just identifying the co-occurrence of substance use, mental health challenges and domestic and family violence, towards a fuller understanding of how they intersect (Bostock et al., 2018).

Despite training and support, Tsantefski et al. (2021) found that, although practitioners valued an approach that focused on making perpetrator action more visible in child protection, they still reported fear and feeling underprepared for work with perpetrators. Nonetheless, they reported confidence in tracking and sharing patterns of perpetrator behaviour and found this helpful in supporting safeguarding for child survivors. McCarry et al. (2021) found that practitioners in early help services identified significant variation in the needs of families where the perpetrator was still living with the family, where there was post-separation abuse or conflict over child contact, and where children had suffered emotional harm resulting from living with domestic violence.

### What Evidence of Effectiveness is There in Organisational Responses to Domestic Violence and Abuse in the Context of Child Protection?

Literature on effectiveness of organisational responses is at an early stage. The research projects evaluating the effectiveness of the various interagency collaborative models reported are based primarily on descriptive and qualitative methodologies. The reviewed studies typically describe the key features of the interventions, their histories, and the processes involved in their development and implementation.

There is descriptive evidence of the enabling factors within organisations that facilitate the embedding of models like Safe and Together. A key enabler to organisational change is a supportive environment, including substantial senior management support, domestic violence and abuse informed and child-focused policies and procedures, and training to increase practitioner skills and confidence (Humphreys & Healey, 2017). Humphreys et al. (2018a) highlight that effective information sharing and multi-agency collaboration was found where there were a number of key factors, including top-down endorsement, stability of leadership, availability of funding, and a shared language. However, further research is needed to confirm these results. (Humphreys et al., 2020).

Humphreys et al. (2020) found that the transformation of practitioner assessment and responses depended on an authorising environment that embeds domestic abuse informed and child-focused policies, procedures and training. The training itself needed to focus on building both skills and confidence. They found that when these provisions were in place, effective change was enabled by an all-of-family approach to practice. Mere training of frontline professionals was not sufficient to bring about a whole system change. This needed to be enacted at all levels of the organisational hierarchy and required cross-sectoral buy-in to enable true service transformation (Humphreys et al., 2020). Social work practice could not fully transform in isolation from other service responses to victim-survivors of domestic violence and abuse unless an effective change has occurred as a result of organisations structuring an all-family approach into practice. However, the authors concluded that there is a long way to go across all sectors to re-orient service systems to the principles of the Safe & Together model.

Some of the reviewed studies examined barriers to effective implementation of the model. For example, work with minoritised communities was hampered by a lack of recognition of their needs and difficulties integrating cultural awareness and domestic violence and abuse expertise (Tsantefski, et al., 2018). Practitioners in one study reported that limited capacity and the volume of cases could compromise the safety of women and children (Tsantefski et al., 2021). Practitioners reported that training in the interagency model itself was not new, but that it did support a shift towards more detailed information sharing, and more regular meetings to discuss high-risk families (Tsantefski et al., 2021).

Working in a multi-agency context is often seen as challenging. Different services hold different “lenses” through which they examine cases, such as a therapeutic, a feminist, or a criminal justice lens, and sometimes sharing a common language is not enough to sustain a unified response (Tsantefski et al., 2018). Practitioners expressed concerns about maintaining confidentiality, particularly in high-risk cases, where careless information sharing could have unintended consequences for families, or impact relationship building if there were misunderstandings in the way information is used (Humphreys & Healey, 2017; Tsantefski et al., 2018; McCarry et al., 2021; Tsantefski et al., 2021). In one study, practitioners in Communities of Practice raised concerns that multi-agency working might have unintended consequences which might reduce effectiveness of interventions and observed that some systems or institutional practices could compound trauma for women and children (Heward-Belle, et al., 2020). For instance, in research on Safe and Together, it was noted that safety was compromised by inadequate or delayed communication and collaboration between organisations or practitioners (Tsantefski et al., 2021). Threats to child protection workers’ or women’s safety led to reluctance to provide information to the police in high-risk cases, which compromised “perpetrator mapping” efforts (Tsantefski et al., 2021).

One article reported on the introduction of a Multi-Agency Triage to introduce a more structured response to disclosures. This enabled a differential response, based on assessment of risk of serious harm. Lower risk cases were signposted to community-based services, whilst more serious cases required statutory intervention (Humphreys et al., 2018b). Such innovation at a policy level had a positive impact in practice, reducing police reports for cases with less serious harm and increasing signposting to non-statutory services (Humphreys et al., 2018b).

#### Impact on Child and Adult Victim-Survivors of Domestic Violence and Abuse

The majority of the included studies did not involve direct engagement with children and parents about the experience of the model. Only two studies considered the perspectives of mothers and children alongside staff’s views (Humphreys et al., 2020; McCarry et al., 2021), and one included fathers’ perspectives (Humphreys et al., 2020). The remaining papers focussed exclusively on practitioners’, managers’, and stakeholders’ experiences and case files.

Evidence around whether practitioner responses to children have been informed by these innovative models is mixed. According to Humphreys and colleagues (2020), practitioners trained in the Safe & Together model are more focussed on developing strategies for partnering with mothers and engaging with fathers rather than keeping a direct focus on children, which sounds almost paradoxical, given that the model revolves around improving parenting practices for children’s wellbeing. However, it is felt that attention to children’s needs and rights in the context of disclosure of domestic violence and abuse is growing. Such a finding is backed by the evaluation of Tsantefski and colleagues (2018), although they reported that often police and child safety practitioners attend directly to the mother’s needs as a means of ensuring children’s safety, which could, conversely, overshadow the child’s views. Unfortunately, many studies testify to the marginal role of children, as they are either portrayed as passive victims of family abuse or are virtually used as “tools” to change the abusive parent’s behaviour, particularly within the Safe & Together Model (Humphreys & Healey, 2017; Humphreys et al., 2018b; O’Leary et al., 2018; Sen et al., 2018).

In terms of changes for families, some studies reported anecdotal evidence of positive practice change linked to the Safe and Together model, framed as more ‘respectful’ communication with families. However, this is based on practitioner self-report (Humphreys et al., 2018a). There is also testimonial evidence (from senior practitioners, team leaders or managers from statutory child protection, non-statutory family services, domestic violence and abuse services, and criminal justice services) that high-risk cases have moved to a lower-risk list (Tsantefski, et al., 2021). In terms of family group conferences, Sen et al., (2018) found evidence from practitioners’ reports that restorative meetings are effective in the creation of “family safety plans” even in high-risk cases, by bringing together the local support networks of both the offending and non-offending parents. For example, Heward-Belle et al.’s (2019) study reported practitioners’ perception that they were partnering more effectively with women survivors to establish safety plans, but this was not more directly assessed with women.

There is evidence from consultations with survivors and with practitioners that children, mothers and fathers appreciate differences in the way practitioners trained in Safe & Together treat them – these practitioners are perceived as more “respectful”. Practitioners recognise that the model’s language not only enables more effective communication with families through a more “mindful” use of questions and “framing” of abusive patterns but also increases the efficiency of interagency communication (Humphreys et al., 2020). Beyond the Communities of Practice, the evaluation of the Gold Coast Domestic Violence Integrated Response shows how child’s safety practitioners shifted their language to reflect the gendered nature of domestic violence and abuse, and the impact that fathers’ behaviour has on children was reportedly being discussed more (Tsantefski et al., 2018). However, none of these studies directly assessed outcomes for adult or child victim-survivors.

A study from Heward-Belle and colleagues (2020) evidences the critical aspects of a “siloed” response to the victim-survivor and the perpetrator of their abuse. It has been noted that mental health practitioners, and also some social workers, tend to lack analysis of overarching social structures embedding policy and practice, failing to recognise sexist or racist biases. Tsantefski and colleagues (2021) also acknowledge that courts are still largely unsuccessful at understanding the protective measures that non-abusive parents take in response to abusive behaviour, substantially impairing the effectiveness of conjoined efforts. This impacts on the capacity of practitioners to partner effectively with adult victim-survivors.

Stevens et al. (2019) found that it was important to consider the impact of narrow eligibility criteria in responding to child experiences of domestic violence and abuse. They argued that focusing just on children who meet ‘high risk’ eligibility criteria meant that the programme was too restrictive and risked overlooking children who appeared to be coping well, through an overemphasis on specific trauma symptoms. The subsequent expansion of eligibility to more families was viewed universally as positive, allowing for the identification and potential engagement of more families in needed services. It was also recognised that there was an issue in terms of the caregiver-selection of children for screening for PTSD, which might have privileged the screening of children who “appeared” most distressed to them.

Assessing the value of a Multi-Agency Triage approach to responding to child protection needs for those impacted by domestic violence and abuse, Humphreys et al. (2018b) found that the approach increased practitioners’ ability to assess risk effectively, to share information more easily, and to respond more appropriately and speedily to the needs of child and adult victim-survivors. Some of these enhancements might seem obvious but are crucial to effective service responses. For instance, they found that the MAT approach meant that receiving agencies could receive more accurate information from referring police officers about crucial details like whether the adult survivors had children.

#### Engagement with Perpetrators

Sen et al. (2018)reported evidence of better perpetrator engagement with family safety planning in family group conferences and suggested that these processes could be a means to restore “personhood to fathers” according to the concept of “reintegrative shaming” that this approach draws from (Braithwaite, 1989). The restorative approach is seen to enable families to voice their concerns and enables effective “participation” of the perpetrator in the decisions, as they are confronted directly before any decisions are made. Coordinators’ anxieties about working with perpetrators are still high (Sen et al., 2018). Similar findings are reported in the Safe and Together evaluations (Tsantefski et al., 2021), which noted better engagement with perpetrators, and enhanced communication in relation to both respect and accountability. However, this is largely based on practitioner report, and as noted above, practitioners still reported some anxiety about this element of the work (Heward-Belle et al., 2019). It was also noted in some research that engagement with the most dangerous perpetrators is still avoided (Tsantefski et al., 2021).

In early help services in the United Kingdom, the research reported that practitioners were more attuned to differences in needs where the perpetrator was still living with the family, where there was post-separation abuse or conflict over child contact, and where children had suffered emotional harm resulting from living with domestic violence (McCarry et al., 2021).

However, in some contexts, caution was expressed by researchers, who noted that change in practice is still in its infancy, particularly with regards to “pivoting to the perpetrator” and integrating adult-focussed practice with children and their needs (Humphreys et al., 2020).

#### Assessing the State of the Evidence

We were unable to locate any outcomes study or process evaluation associated with any of the intervention models reviewed. The research reviewed here is largely concerned with practitioner perspectives and primarily deploys qualitative methods.

The reviewed studies indicated a need for assessment of behaviour change (particularly of perpetrators) but did not provide clear recommendations on how to measure such change and did not directly evidence such change. Similarly, there is no objective data on enhanced outcomes for adult or child victim-survivors. Rather it is assumed that better communication, better partnerships with adult victim-survivors and increased accountability for perpetrators will improve outcomes. Whilst this may well be the case, it is not evidenced in the research reviewed.

One study (Humphreys et al., 2018a) deployed a ‘case reading’ approach to evaluation, examining case files for evidence that workers were: building positive partnerships with adult victim-survivors; holding the perpetrator accountable; and making visible their pattern of abusive behaviour. This provides useful documentation of the practice transformation enabled by the introduction of the model, but does not offer insight into the impact of this for the families worked with.

In Australia, multidisciplinary “communities of practice” were utilised to assess the impact of the Safe and Together model (Heward-Belle et al., 2020; Tsantefski et al., 2021). These “communities of practice” are a way for practitioners to share knowledge and acquire skills by working collectively and regularly on a shared problem or challenge. As with the case reading methods, this research provides rich data and insights into how practice is transformed, supported by robust and rigorous methods of qualitative data collection and analysis. However, it is limited to practitioner voice, and does not evidence either the experiences or outcomes for children, adult victim-survivors, or perpetrators.

## Discussion

This paper has sought to update knowledge about the range of approaches being used in relation to interagency work regarding child protection and domestic violence and abuse. Additionally, we have sought to also explore what the published literature says about professionals’ understanding and responses to women, children and men experiencing domestic violence and abuse; the impact of the interventions on improving professionals’ assessment of and responses to the disclosure of domestic violence and abuse; and the evidence of effectiveness of these models in transforming services and practice.

It is clear that the increased recognition of the impact of domestic violence and abuse on children, and the unintended, but nonetheless harmful impacts of the child protection response on victims/survivors, has resulted in new ways of working being developed and studied. The discourse on mother’s failure to protect has resulted in many women feeling blamed for the abuse that they and their children experience, while simultaneously being ostracised from the support they require (Nixon et al., 2017). Such a discourse has also had the unintended effect of invisiblising men, and reducing the development of interventions to address their abusive behaviour (Devaney & Lazenbatt, 2016). The failure of professionals to engage with abusive men simultaneously enhances perpetrators’ ability to seem immune from the need to change and to exert even greater control and domination of their family.

The research reviewed here documents organisational and individual practitioner level changes to address the above issues and to enhance partnership working with adult victim-survivors. However, research on these models is still very much in its infancy. There remain significant gaps in the evidence base for the models reviewed in this study. In particular, the outcomes for child and adult victim-survivors, and for perpetrators remains unassessed. Indeed, the reviewed research does not recommend how such outcomes should be appropriately assessed. There is also no evidence in the literature reviewed here, or in the earlier Macvean et al. (2018) study, of how the introduction of these models impacts pathways through services, or destinations beyond services. An outcomes and process evaluation is therefore a pressing need to enable confident statements about the effectiveness of these promising interventions. More robust studies are required to ascertain the specific needs and supports required by child and adult victims and perpetrators, and to determine the actual effectiveness of new approaches to immediate and longer-term safeguarding. The ability of services to flex their delivery model in response to the needs of families is essential for supporting the engagement of, and fostering a sense of control for, families receiving support. There is a growing evidence base about some models, such as Safe & Together, that is rooted in strong partnerships between model developers, agencies using the model and independent researchers, which increases the likelihood of a more robust and comprehensive evidence base in the future; however, there is still no structured assessment of the impact of this model on families’ wellbeing and perpetrators’ accountability. Further research should seek to gather the voices of service users themselves, including the voices of perpetrator parents.

It was also noted that most of the studies did not include the experiences of the people for whom the services were being developed. Instead, changes in practices are documented solely through the lens of practitioner and manager views. This is a significant limitation in the evidence base. In addition, it was difficult to trace through the literature whether there had been any involvement of children and young people or adult victims-survivors, in the development and implementation of the models assessed. Based on the evidence reviewed, it appears that practitioners and agencies have developed their models and interventions as an alternative to previous ways of working, without direct participation. The models themselves clearly take into account practitioner perspectives on the failings of previous ways of working, but without direct participation from the intended beneficiaries, it seems likely these models will still contain blind spots about the perspectives and experiences of victim/survivors (and indeed of perpetrators) with child protection involvement. There is also a real danger that new approaches may perpetuate the racialised biases inherent in current approaches (Kelly et al., 2022), without engagement with impacted communities. This could reflect a paternalistic attitude to the development of new interventions and models, rooted in traditional ways of working that privilege certain types of knowledge as necessary and sufficient, and relegating other types of knowledge, such as lived experience, as being anecdotal and unscientific.

The absence of children’s perspectives in the research raises concerns that in seeking to address the criticism of the negative impact of child protection responses on mothers, the needs of children may have received less consideration. It is important to recognise that, whilst the needs of children and mothers are often connected, they are also distinct, and can diverge (Buchanan & Moulding, 2021). More than a decade of research has convincingly evidenced the importance of recognising children’s experiences of domestic violence and abuse in their own right, and demonstrates the value of direct engagement with children and young people in understanding their unique perspective, and gaining their insights into how their needs are best met (for example, Callaghan et al., 2019; Houghton, 2017; Morrison et al., 2020). This appears to reiterate the issues identified in Hester’s (2011) ‘Three Planet model’, in which she highlighted the disconnect between domestic violence and abuse, perpetrator and child protection services. In that paper, Hester (2011) noted that the differing orientations of the child protection and domestic violence and abuse fields have resulted in friction between systems that should have mutual aims, but are often out of step. In seeking to develop new approaches, the potential is for the needs of children to be understood through a particular lens, and there is a risk that a feminist paradigm might subsume children’s needs within those of mothers. It is important therefore that interventions that are oriented towards protecting and supporting children should incorporate both a feminist *and* a child protection lens, to ensure that all victim-survivors are represented, understood and engaged appropriately in services.

The models assessed in the research reviewed here appear to work from an assumption that interagency / multiagency collaboration is a necessary force for good. This is perhaps unsurprising since it is the frequent recommendation of serious case and domestic homicide reviews (Department for Education, 2022). However, some of the studies reviewed have cautioned that communication between agencies might be associated with unintended and negative outcomes for victim-survivors. Whilst these cautions were tentative in the papers, nonetheless they do appear to warrant further attention. In particular, where different agencies may have varying priorities and systems, there seems to be some potential for conflict, and it is also important to consider how competitive tendering processes that typify the way many services are funded might be hostile to collaborative endeavours.

### Limitations

This review used a scoping review approach, applying systematic methods to search for and evaluate select studies. Some limits were placed on methodology: only English language papers were included, books, theses, and conference papers were not included, and authors were not contacted for additional studies and data. Consequently, some relevant work may have been missed.

## Conclusion

There is a growing acceptance of the need to deliver child protection services in ways that can promote the ongoing safety of child and adult victims of domestic violence and abuse, while holding individuals who use violence and abuse within their familial relationships accountable for their behaviour and its impact. While the number of interagency models and interventions is increasing, the evidence base is still growing in respect of the utility and effectiveness of such new ways of working. Their capacity to improve outcomes such as safety and wellbeing of adult and child victims still requires investigation.

## Supplementary information

Below is the link to the electronic supplementary material.

ESM 1 (DOCX 36.5 KB)

## Data Availability

The data relating to searches and identified literature are available from the corresponding author.

## Footnotes

1 In this study “domestic abuse” is defined as a pattern of violence and abusive or coercive behaviour in a current or former intimate partnership, used to gain or maintain power and control. It may also be termed domestic violence or intimate partner violence.
