# Supplementary material for: The Child Protection Response to Domestic Violence and Abuse: a Scoping Review of Interagency Interventions, Models and Collaboration
Source: J Fam Violence. 2024 Feb 19;41(4):653–73. doi: 10.1007/s10896-024-00681-4 (PMC13198489; doi:10.1007/s10896-024-00681-4)
Supplement: Supplementary file 1 — (DOCX 36.5 KB) [file 10896_2024_681_MOESM1_ESM.docx]

Records were downloaded 23/07/21, with the exception of ASSIA and Social Services Abstracts which were downloaded on 03 and 04 August respectively. This was due to a daily limit on the number of exports from the ProQuest platform.

Search terms were developed using the original search strategy from Macvean, M. L. et al. (2018) Facilitating the Collaborative Interface between Child Protection and Specialist Domestic Violence Services: A Scoping Review. *Australian social work*. 71 (2), 148–161.

Searches were tested for recall in the PsycInfo database (EBSCOhost) using gold-standard indicator papers.

A database limit of year of publication from 2015 was applied, to retrieve records added to databases after searches from Macvean et al (2018) were conducted.

| # | Database | Search terms | Field | Limits | Date | Results |
| --- | --- | --- | --- | --- | --- | --- |
| 1 | **PsycINFO via Ovid** | 1. ((domestic or family or interpersonal or intimate or Intimate partner or ex-partner or spous* or wife or relationship or couple* or partner or marital) adj3 (violen* or abus* or conflict*)).mp.  2. (femicide or batterer or battered wom* or battered wi* or battered spouse* or battered partner* or battered female*).mp.  3. (women* specialist service* or victim support service* or men* specialist service* or perpetrator program* or men* behaviour change program*).mp.  4. (child protect* or child welfare or CPS or ((infan* or child* or minor* or toddler* or baby or babies or adolescent* or teen* or young person or youth or young people) adj3 (maltreat* or neglect* or abuse*))).mp. [mp=title, abstract, heading word, table of contents, key concepts, original title, tests & measures, mesh]  5. (foster care or group home or group care or residential care or congregate care or kinship care or relative care or customary care or shelter care or temporary care or looked after child* or child place* or place* in care or out-of-home care or out of home care or foster child* or foster youth).mp.  6. (((collaborat* or coalition* or network* or cooperat* or integrate* or partnership* or link* or coordinat* or streamlin* or work* or initiative* or align* or attune*) adj3 (work* or approach* or service* or practice* or care or intervention* or system* or initiative* or area-based or area based or locally-based or locally based or place-based or place based or community based or community-based or community response* or inter-agency or interagency or multiagency or multi-agency or multisite or multi-site)) or (coordinating council* or colocat* or co-locat*)).mp. [mp=title, abstract, heading word, table of contents, key concepts, original title, tests & measures, mesh]  7. (randomi* or random* control* or RCT or clinical trial* or control group* or evaluation stud* or study design or double-blind or placebo or meta-anal* or meta anal* or metaanal* or systematic Review* or scoping review* or econometric or propensity score matching or Heckman* or instrumental variable* or natural experiment or Bayesian or comparison group* or treat* group* or wait* list* or wait*-list* or control* condition* or quasi-ex* or quasiex*or evaluation* or case control* or case-control* or cross sectional or cross-sectional or case study or case studies or synthesis of studies or study synthesis or evaluation* or studies or study or research or investigation* or trial* or statistical* significan*).mp.  8. 1 or 2 or 3  9. 4 or 5  10. 6 and 7 and 8 and 9  11. limit 10 to yr="2015 -Current" | mp | Date 2015-current | 23/07/2021 | 433 |
| 2 | **MEDLINE(R) via Ovid** | 1. ((domestic or family or interpersonal or intimate or Intimate partner or ex-partner or spous* or wife or relationship or couple* or partner or marital) adj3 (violen* or abus* or conflict*)).mp.  2. (femicide or batterer or battered wom* or battered wi* or battered spouse* or battered partner* or battered female*).mp.  3. (women* specialist service* or victim support service* or men* specialist service* or perpetrator program* or men* behaviour change program*).mp.  4. (child protect* or child welfare or CPS or ((infan* or child* or minor* or toddler* or baby or babies or adolescent* or teen* or young person or youth or young people) adj3 (maltreat* or neglect* or abuse*))).mp. [mp=title, abstract, original title, name of substance word, subject heading word, floating sub-heading word, keyword heading word, organism supplementary concept word, protocol supplementary concept word, rare disease supplementary concept word, unique identifier, synonyms]  5. (foster care or group home or group care or residential care or congregate care or kinship care or relative care or customary care or shelter care or temporary care or looked after child* or child place* or place* in care or out-of-home care or out of home care or foster child* or foster youth).mp.  6. (((collaborat* or coalition* or network* or cooperat* or integrate* or partnership* or link* or coordinat* or streamlin* or work* or initiative* or align* or attune*) adj3 (work* or approach* or service* or practice* or care or intervention* or system* or initiative* or area-based or area based or locally-based or locally based or place-based or place based or community based or community-based or community response* or inter-agency or interagency or multiagency or multi-agency or multisite or multi-site)) or (coordinating council* or colocat* or co-locat*)).mp. [mp=title, abstract, original title, name of substance word, subject heading word, floating sub-heading word, keyword heading word, organism supplementary concept word, protocol supplementary concept word, rare disease supplementary concept word, unique identifier, synonyms]  7. (randomi* or random* control* or RCT or clinical trial* or control group* or evaluation stud* or study design or double-blind or placebo or meta-anal* or meta anal* or metaanal* or systematic Review* or scoping review* or econometric or propensity score matching or Heckman* or instrumental variable* or natural experiment or Bayesian or comparison group* or treat* group* or wait* list* or wait*-list* or control* condition* or quasi-ex* or quasiex*or evaluation* or case control* or case-control* or cross sectional or cross-sectional or case study or case studies or synthesis of studies or study synthesis or evaluation* or studies or study or research or investigation* or trial* or statistical* significan*).mp.  8. 1 or 2 or 3  9. 4 or 5  10. 6 and 7 and 8 and 9  11. limit 10 to yr="2015 -Current" | mp | Date 2015-current | 23/07/21 | 214 |
| 3 | **Embase via Ovid** | 1. ((domestic or family or interpersonal or intimate or Intimate partner or ex-partner or spous* or wife or relationship or couple* or partner or marital) adj3 (violen* or abus* or conflict*)).mp.  2. (femicide or batterer or battered wom* or battered wi* or battered spouse* or battered partner* or battered female*).mp.  3. (women* specialist service* or victim support service* or men* specialist service* or perpetrator program* or men* behaviour change program*).mp.  4. (child protect* or child welfare or CPS or ((infan* or child* or minor* or toddler* or baby or babies or adolescent* or teen* or young person or youth or young people) adj3 (maltreat* or neglect* or abuse*))).mp. [mp=title, abstract, heading word, drug trade name, original title, device manufacturer, drug manufacturer, device trade name, keyword, floating subheading word, candidate term word]  5. (foster care or group home or group care or residential care or congregate care or kinship care or relative care or customary care or shelter care or temporary care or looked after child* or child place* or place* in care or out-of-home care or out of home care or foster child* or foster youth).mp.  6. (((collaborat* or coalition* or network* or cooperat* or integrate* or partnership* or link* or coordinat* or streamlin* or work* or initiative* or align* or attune*) adj3 (work* or approach* or service* or practice* or care or intervention* or system* or initiative* or area-based or area based or locally-based or locally based or place-based or place based or community based or community-based or community response* or inter-agency or interagency or multiagency or multi-agency or multisite or multi-site)) or (coordinating council* or colocat* or co-locat*)).mp. [mp=title, abstract, heading word, drug trade name, original title, device manufacturer, drug manufacturer, device trade name, keyword, floating subheading word, candidate term word]  7. (randomi* or random* control* or RCT or clinical trial* or control group* or evaluation stud* or study design or double-blind or placebo or meta-anal* or meta anal* or metaanal* or systematic Review* or scoping review* or econometric or propensity score matching or Heckman* or instrumental variable* or natural experiment or Bayesian or comparison group* or treat* group* or wait* list* or wait*-list* or control* condition* or quasi-ex* or quasiex*or evaluation* or case control* or case-control* or cross sectional or cross-sectional or case study or case studies or synthesis of studies or study synthesis or evaluation* or studies or study or research or investigation* or trial* or statistical* significan*).mp.  8. 1 or 2 or 3  9. 4 or 5  10. 6 and 7 and 8 and 9  11. limit 10 to yr="2015 -Current" | mp | Date 2015-current | 23/07/21 | 409 |
|  | **CINAHL via EBSCOhost** | ((((violen* or abus* or conflict*) N3 domestic) or ((violen* or abus* or conflict*) N3 family) or ((violen* or abus* or conflict*) N3 interpersonal) or ((violen* or abus* or conflict*) N3 intimate) or ((violen* or abus* or conflict*) N3 intimate partner) or ((violen* or abus* or conflict*) N3 ex-partner) or ((violen* or abus* or conflict*) N3 spouse*) or ((violen* or abus* or conflict*) N3 wife) or ((violen* or abus* or conflict*) N3 relationship) or ((violen* or abus* or conflict*) N3 couple*) or ((violen* or abus* or conflict*) N3 partner) or ((violen* or abus* or conflict*) N3 marital) or (femicide or batterer or battered wom* or battered wi* or battered spouse* or battered partner* or battered female*) or ((women* specialist service*) or (victim support service*) or (men* specialist service*) or (perpetrator program*) or (men* behaviour change program*)))  AND  ((child protect*) OR (child welfare) or (CPS) OR ((maltreat* or neglect* or abuse*) N3 infan*) or ((maltreat* or neglect* or abuse*) N3 child*) or ((maltreat* or neglect* or abuse*) N3 minor*) or ((maltreat* or neglect* or abuse*) N3 toddler*) or ((maltreat* or neglect* or abuse*) N3 baby) or ((maltreat* or neglect* or abuse*) N3 babies) or ((maltreat* or neglect* or abuse*) N3 adolescent*) or ((maltreat* or neglect* or abuse*) N3 teen*) or ((maltreat* or neglect* or abuse*) N3 young person) or ((maltreat* or neglect* or abuse*) N3 youth) or ((maltreat* or neglect* or abuse*) N3 young people) OR (foster care or group home or group care or residential care or congregate care or kinship care or relative care or customary care or shelter care or temporary care or looked after child* or child place* or place* in care or out-of-home care or out of home care or foster child* or foster youth))  AND  (((collaborat* or coalition* or network* or cooperat* or integrate* or partnership* or link* or coordinat* or streamlin* or work* or initiative* or align* or attune*) AND (work* or approach* or service* or practice* or care or intervention* or system* or initiative* or area-based or area based or locally-based or locally based or place-based or place based or community based or community-based or community response* or inter-agency or interagency or multiagency or multi-agency or multisite or multi-site)) OR ((coordinating council*) or (colocat*) or (co-locat*)))  AND  ((randomi* OR random* control* OR RCT OR clinical trial* OR control group* OR evaluation stud* OR study design OR double-blind OR placebo OR meta-anal* OR meta anal* OR metaanal* OR systematic Review* OR econometric OR propensity score matching OR Heckman* OR instrumental variable* OR natural experiment OR Bayesian or comparison group* or treat* group* or wait* list* or wait*-list* or control* condition* or quasi-ex* or quasiex*or evaluation* or case control* or case-control* or cross sectional or cross-sectional or case study or case studies or synthesis of studies or study synthesis or evaluation* or studies or study or research or investigation* or trial* or statistical* significan*))) | AB  Boolean/Phrase search | Date 2015-current | 23/07/21 | 883 |
|  | **Criminal Justice Abstracts via Ebsco** | ((((violen* or abus* or conflict*) N3 domestic) or ((violen* or abus* or conflict*) N3 family) or ((violen* or abus* or conflict*) N3 interpersonal) or ((violen* or abus* or conflict*) N3 intimate) or ((violen* or abus* or conflict*) N3 intimate partner) or ((violen* or abus* or conflict*) N3 ex-partner) or ((violen* or abus* or conflict*) N3 spouse*) or ((violen* or abus* or conflict*) N3 wife) or ((violen* or abus* or conflict*) N3 relationship) or ((violen* or abus* or conflict*) N3 couple*) or ((violen* or abus* or conflict*) N3 partner) or ((violen* or abus* or conflict*) N3 marital) or (femicide or batterer or battered wom* or battered wi* or battered spouse* or battered partner* or battered female*) or ((women* specialist service*) or (victim support service*) or (men* specialist service*) or (perpetrator program*) or (men* behaviour change program*))) AND ((child protect*) OR (child welfare) or (CPS) OR ((maltreat* or neglect* or abuse*) N3 infan*) or ((maltreat* or neglect* or abuse*) N3 child*) or ((maltreat* or neglect* or abuse*) N3 minor*) or ((maltreat* or neglect* or abuse*) N3 toddler*) or ((maltreat* or neglect* or abuse*) N3 baby) or ((maltreat* or neglect* or abuse*) N3 babies) or ((maltreat* or neglect* or abuse*) N3 adolescent*) or ((maltreat* or neglect* or abuse*) N3 teen*) or ((maltreat* or neglect* or abuse*) N3 young person) or ((maltreat* or neglect* or abuse*) N3 youth) or ((maltreat* or neglect* or abuse*) N3 young people) OR (foster care or group home or group care or residential care or congregate care or kinship care or relative care or customary care or shelter care or temporary care or looked after child* or child place* or place* in care or out-of-home care or out of home care or foster child* or foster youth)) AND (((collaborat* or coalition* or network* or cooperat* or integrate* or partnership* or link* or coordinat* or streamlin* or work* or initiative* or align* or attune*) AND (work* or approach* or service* or practice* or care or intervention* or system* or initiative* or area-based or area based or locally-based or locally based or place-based or place based or community based or community-based or community response* or inter-agency or interagency or multiagency or multi-agency or multisite or multi-site)) OR ((coordinating council*) or (colocat*) or (co-locat*))) AND ((randomi* OR random* control* OR RCT OR clinical trial* OR control group* OR evaluation stud* OR study design OR double-blind OR placebo OR meta-anal* OR meta anal* OR metaanal* OR systematic Review* OR scoping review* OR econometric OR propensity score matching OR Heckman* OR instrumental variable* OR natural experiment OR Bayesian or comparison group* or treat* group* or wait* list* or wait*-list* or control* condition* or quasi-ex* or quasiex*or evaluation* or case control* or case-control* or cross sectional or cross-sectional or case study or case studies or synthesis of studies or study synthesis or evaluation* or studies or study or research or investigation* or trial* or statistical* significan*))) | AB  Boolean/Phrase search | Date 2015-current |  |  |
|  | **ERIC via Ebsco host** | ((((violen* or abus* or conflict*) N3 domestic) or ((violen* or abus* or conflict*) N3 family) or ((violen* or abus* or conflict*) N3 interpersonal) or ((violen* or abus* or conflict*) N3 intimate) or ((violen* or abus* or conflict*) N3 intimate partner) or ((violen* or abus* or conflict*) N3 ex-partner) or ((violen* or abus* or conflict*) N3 spouse*) or ((violen* or abus* or conflict*) N3 wife) or ((violen* or abus* or conflict*) N3 relationship) or ((violen* or abus* or conflict*) N3 couple*) or ((violen* or abus* or conflict*) N3 partner) or ((violen* or abus* or conflict*) N3 marital) or (femicide or batterer or battered wom* or battered wi* or battered spouse* or battered partner* or battered female*) or ((women* specialist service*) or (victim support service*) or (men* specialist service*) or (perpetrator program*) or (men* behaviour change program*))) AND ((child protect*) OR (child welfare) or (CPS) OR ((maltreat* or neglect* or abuse*) N3 infan*) or ((maltreat* or neglect* or abuse*) N3 child*) or ((maltreat* or neglect* or abuse*) N3 minor*) or ((maltreat* or neglect* or abuse*) N3 toddler*) or ((maltreat* or neglect* or abuse*) N3 baby) or ((maltreat* or neglect* or abuse*) N3 babies) or ((maltreat* or neglect* or abuse*) N3 adolescent*) or ((maltreat* or neglect* or abuse*) N3 teen*) or ((maltreat* or neglect* or abuse*) N3 young person) or ((maltreat* or neglect* or abuse*) N3 youth) or ((maltreat* or neglect* or abuse*) N3 young people) OR (foster care or group home or group care or residential care or congregate care or kinship care or relative care or customary care or shelter care or temporary care or looked after child* or child place* or place* in care or out-of-home care or out of home care or foster child* or foster youth))  AND  (((collaborat* or coalition* or network* or cooperat* or integrate* or partnership* or link* or coordinat* or streamlin* or work* or initiative* or align* or attune*) AND (work* or approach* or service* or practice* or care or intervention* or system* or initiative* or area-based or area based or locally-based or locally based or place-based or place based or community based or community-based or community response* or inter-agency or interagency or multiagency or multi-agency or multisite or multi-site)) OR ((coordinating council*) or (colocat*) or (co-locat*)))  AND  ((randomi* OR random* control* OR RCT OR clinical trial* OR control group* OR evaluation stud* OR study design OR double-blind OR placebo OR meta-anal* OR meta anal* OR metaanal* OR systematic Review* OR econometric OR propensity score matching OR Heckman* OR instrumental variable* OR natural experiment OR Bayesian or comparison group* or treat* group* or wait* list* or wait*-list* or control* condition* or quasi-ex* or quasiex*or evaluation* or case control* or case-control* or cross sectional or cross-sectional or case study or case studies or synthesis of studies or study synthesis or evaluation* or studies or study or research or investigation* or trial* or statistical* significan*))) | AB  Boolean/Phrase search | Date 2015-current | 23/07/21 | 55 |
|  | **ASSIA via Proquest** | AB((((violen* or abus* or conflict*) NEAR/3 domestic) or ((violen* or abus* or conflict*) NEAR/3 family) or ((violen* or abus* or conflict*) NEAR/3 interpersonal) or ((violen* or abus* or conflict*) NEAR/3 intimate) or ((violen* or abus* or conflict*) NEAR/3 intimate partner) or ((violen* or abus* or conflict*) NEAR/3 ex-partner) or ((violen* or abus* or conflict*) NEAR/3 spouse*) or ((violen* or abus* or conflict*) NEAR/3 wife) or ((violen* or abus* or conflict*) NEAR/3 relationship) or ((violen* or abus* or conflict*) NEAR/3 couple*) or ((violen* or abus* or conflict*) NEAR/3 partner) or ((violen* or abus* or conflict*) NEAR/3 marital) or (femicide or batterer or battered wom* or battered wi* or battered spouse* or battered partner* or battered female*) or ((women* specialist service*) or (victim support service*) or (men* specialist service*) or (perpetrator program*) or (men* behaviour change program*))) AND ((child protect*) OR (child welfare) or (CPS) OR ((maltreat* or neglect* or abuse*) NEAR/3 infan*) or ((maltreat* or neglect* or abuse*) NEAR/3 child*) or ((maltreat* or neglect* or abuse*) NEAR/3 minor*) or ((maltreat* or neglect* or abuse*) NEAR/3 toddler*) or ((maltreat* or neglect* or abuse*) NEAR/3 baby) or ((maltreat* or neglect* or abuse*) NEAR/3 babies) or ((maltreat* or neglect* or abuse*) NEAR/3 adolescent*) or ((maltreat* or neglect* or abuse*) NEAR/3 teen*) or ((maltreat* or neglect* or abuse*) NEAR/3 young person) or ((maltreat* or neglect* or abuse*) NEAR/3 youth) or ((maltreat* or neglect* or abuse*) NEAR/3 young people) OR (foster care or group home or group care or residential care or congregate care or kinship care or relative care or customary care or shelter care or temporary care or looked after child* or child place* or place* in care or out-of-home care or out of home care or foster child* or foster youth)) AND (((collaborat* or coalition* or network* or cooperat* or integrate* or partnership* or link* or coordinat* or streamlin* or work* or initiative* or align* or attune*) AND (work* or approach* or service* or practice* or care or intervention* or system* or initiative* or area-based or area based or locally-based or locally based or place-based or place based or community based or community-based or community response* or inter-agency or interagency or multiagency or multi-agency or multisite or multi-site)) OR ((coordinating council*) or (colocat*) or (co-locat*))) AND ((randomi* OR random* control* OR RCT OR clinical trial* OR control group* OR evaluation stud* OR study design OR double-blind OR placebo OR meta-anal* OR meta anal* OR metaanal* OR systematic Review* OR scoping review* OR econometric OR propensity score matching OR Heckman* OR instrumental variable* OR natural experiment OR Bayesian or comparison group* or treat* group* or wait* list* or wait*-list* or control* condition* or quasi-ex* or quasiex*or evaluation* or case control* or case-control* or cross sectional or cross-sectional or case study or case studies or synthesis of studies or study synthesis or evaluation* or studies or study or research or investigation* or trial* or statistical* significan*))) | AB | Date 2015-current | 03/08/2021 | 382 |
|  | **Sociological Abstracts via Proquest** | AB((((violen* or abus* or conflict*) NEAR/3 domestic) or ((violen* or abus* or conflict*) NEAR/3 family) or ((violen* or abus* or conflict*) NEAR/3 interpersonal) or ((violen* or abus* or conflict*) NEAR/3 intimate) or ((violen* or abus* or conflict*) NEAR/3 intimate partner) or ((violen* or abus* or conflict*) NEAR/3 ex-partner) or ((violen* or abus* or conflict*) NEAR/3 spouse*) or ((violen* or abus* or conflict*) NEAR/3 wife) or ((violen* or abus* or conflict*) NEAR/3 relationship) or ((violen* or abus* or conflict*) NEAR/3 couple*) or ((violen* or abus* or conflict*) NEAR/3 partner) or ((violen* or abus* or conflict*) NEAR/3 marital) or (femicide or batterer or battered wom* or battered wi* or battered spouse* or battered partner* or battered female*) or ((women* specialist service*) or (victim support service*) or (men* specialist service*) or (perpetrator program*) or (men* behaviour change program*))) AND ((child protect*) OR (child welfare) or (CPS) OR ((maltreat* or neglect* or abuse*) NEAR/3 infan*) or ((maltreat* or neglect* or abuse*) NEAR/3 child*) or ((maltreat* or neglect* or abuse*) NEAR/3 minor*) or ((maltreat* or neglect* or abuse*) NEAR/3 toddler*) or ((maltreat* or neglect* or abuse*) NEAR/3 baby) or ((maltreat* or neglect* or abuse*) NEAR/3 babies) or ((maltreat* or neglect* or abuse*) NEAR/3 adolescent*) or ((maltreat* or neglect* or abuse*) NEAR/3 teen*) or ((maltreat* or neglect* or abuse*) NEAR/3 young person) or ((maltreat* or neglect* or abuse*) NEAR/3 youth) or ((maltreat* or neglect* or abuse*) NEAR/3 young people) OR (foster care or group home or group care or residential care or congregate care or kinship care or relative care or customary care or shelter care or temporary care or looked after child* or child place* or place* in care or out-of-home care or out of home care or foster child* or foster youth)) AND (((collaborat* or coalition* or network* or cooperat* or integrate* or partnership* or link* or coordinat* or streamlin* or work* or initiative* or align* or attune*) AND (work* or approach* or service* or practice* or care or intervention* or system* or initiative* or area-based or area based or locally-based or locally based or place-based or place based or community based or community-based or community response* or inter-agency or interagency or multiagency or multi-agency or multisite or multi-site)) OR ((coordinating council*) or (colocat*) or (co-locat*))) AND ((randomi* OR random* control* OR RCT OR clinical trial* OR control group* OR evaluation stud* OR study design OR double-blind OR placebo OR meta-anal* OR meta anal* OR metaanal* OR systematic Review* OR scoping review* OR econometric OR propensity score matching OR Heckman* OR instrumental variable* OR natural experiment OR Bayesian or comparison group* or treat* group* or wait* list* or wait*-list* or control* condition* or quasi-ex* or quasiex*or evaluation* or case control* or case-control* or cross sectional or cross-sectional or case study or case studies or synthesis of studies or study synthesis or evaluation* or studies or study or research or investigation* or trial* or statistical* significan*))) | AB | Date 2015-current | 23/07/21 | 229 |
|  | Social Care Online | violence or abuse or conflict and domestic or family or spouse or partner or intimate or wife or relationship or marital or couple  AND  collaborative or collaboration or collaborate or collaborated or coalition or coalitions or network or networks or cooperative or cooperation or intergrate or integrated or partnership or partnerships or coordinate or coordinated or streamline or streamlined or initiative or initiatives or aligned or aligns or align or attune or attunes or attuned or interagency or mulitagency or "inter-agency" or "multi-agency"  AND  child or children or infant or infants or adolescent or adolescents or youth or young | All fields |  | 23/07/21 | 5 |
|  | Social Services Abstracts via Proquest | AB((((violen* or abus* or conflict*) NEAR/3 domestic) or ((violen* or abus* or conflict*) NEAR/3 family) or ((violen* or abus* or conflict*) NEAR/3 interpersonal) or ((violen* or abus* or conflict*) NEAR/3 intimate) or ((violen* or abus* or conflict*) NEAR/3 intimate partner) or ((violen* or abus* or conflict*) NEAR/3 ex-partner) or ((violen* or abus* or conflict*) NEAR/3 spouse*) or ((violen* or abus* or conflict*) NEAR/3 wife) or ((violen* or abus* or conflict*) NEAR/3 relationship) or ((violen* or abus* or conflict*) NEAR/3 couple*) or ((violen* or abus* or conflict*) NEAR/3 partner) or ((violen* or abus* or conflict*) NEAR/3 marital) or (femicide or batterer or battered wom* or battered wi* or battered spouse* or battered partner* or battered female*) or ((women* specialist service*) or (victim support service*) or (men* specialist service*) or (perpetrator program*) or (men* behaviour change program*))) AND ((child protect*) OR (child welfare) or (CPS) OR ((maltreat* or neglect* or abuse*) NEAR/3 infan*) or ((maltreat* or neglect* or abuse*) NEAR/3 child*) or ((maltreat* or neglect* or abuse*) NEAR/3 minor*) or ((maltreat* or neglect* or abuse*) NEAR/3 toddler*) or ((maltreat* or neglect* or abuse*) NEAR/3 baby) or ((maltreat* or neglect* or abuse*) NEAR/3 babies) or ((maltreat* or neglect* or abuse*) NEAR/3 adolescent*) or ((maltreat* or neglect* or abuse*) NEAR/3 teen*) or ((maltreat* or neglect* or abuse*) NEAR/3 young person) or ((maltreat* or neglect* or abuse*) NEAR/3 youth) or ((maltreat* or neglect* or abuse*) NEAR/3 young people) OR (foster care or group home or group care or residential care or congregate care or kinship care or relative care or customary care or shelter care or temporary care or looked after child* or child place* or place* in care or out-of-home care or out of home care or foster child* or foster youth)) AND (((collaborat* or coalition* or network* or cooperat* or integrate* or partnership* or link* or coordinat* or streamlin* or work* or initiative* or align* or attune*) AND (work* or approach* or service* or practice* or care or intervention* or system* or initiative* or area-based or area based or locally-based or locally based or place-based or place based or community based or community-based or community response* or inter-agency or interagency or multiagency or multi-agency or multisite or multi-site)) OR ((coordinating council*) or (colocat*) or (co-locat*))) AND ((randomi* OR random* control* OR RCT OR clinical trial* OR control group* OR evaluation stud* OR study design OR double-blind OR placebo OR meta-anal* OR meta anal* OR metaanal* OR systematic Review* OR scoping review* OR econometric OR propensity score matching OR Heckman* OR instrumental variable* OR natural experiment OR Bayesian or comparison group* or treat* group* or wait* list* or wait*-list* or control* condition* or quasi-ex* or quasiex*or evaluation* or case control* or case-control* or cross sectional or cross-sectional or case study or case studies or synthesis of studies or study synthesis or evaluation* or studies or study or research or investigation* or trial* or statistical* significan*))) | AB | Date 2015-current | 04/08/21 | 349 |
